# Atomistic Insights into Hydrogen Diffusion and Deformation Mechanisms in FeCrNi-Based Austenitic Stainless Steels: Effects of Alloying, Temperature, and Hydrogen Concentration

**DOI:** 10.3390/molecules31101688

**Published:** 2026-05-16

**Authors:** Jiaqing Li, Zubin Huang, Liang Zhang, Zhiye Zheng, Che Zhang, Shihang Rao, Lin Teng, Lilong Jiang

**Affiliations:** 1 College of Chemical Engineering, Fuzhou University, Fuzhou 350108, China; 2 International Joint Laboratory for Light Alloys (MOE), College of Materials Science and Engineering, Chongqing University, Chongqing 400044, China; 3 Department of Mechanical Engineering, The University of Melbourne, Parkville, VIC 3010, Australia; 4 National Engineering Research Center of Chemical Fertilizer Catalyst, Fuzhou University, Fuzhou 350108, China

**Keywords:** austenitic stainless steel, alloying elements, phase transformation, dislocation plasticity, temperature effect, hydrogen

## Abstract

This study employs molecular dynamics simulations to investigate hydrogen diffusion and deformation mechanisms in FeCrNi-based austenitic stainless steels, with a focus on the effects of alloying composition, temperature, and hydrogen concentration. Arrhenius analysis reveals that Cr increases, while Ni decreases, the activation energy for hydrogen migration. Alloys with low Cr and Ni contents (6 wt.%) promote FCC→BCC→HCP martensitic transformations, accompanied by stress drops, whereas high Cr or Ni levels (24 wt.%) suppress these transformations and favour dislocation plasticity dominated by cross-slip. High hydrogen concentrations reduce stacking-fault energy, activating dense Shockley partial dislocations in agreement with hydrogen-enhanced localised plasticity. Elevated temperatures and high hydrogen concentrations synergistically promote dislocation-mediated plasticity and facilitate vacancy formation, which can cluster into hydrogen–vacancy complexes and proto-nanovoids, accelerating material failure. These findings advance our understanding of the coupled effects of composition, hydrogen, and temperature on degradation in austenitic stainless steels and provide guidance for tailoring Cr/Ni ratios, controlling hydrogen content, and optimising service temperatures in the design of hydrogen-related structural alloys.

## 1. Introduction

Recognised as a key pillar of the global clean energy transition, hydrogen (H) has become a cornerstone of future sustainable energy systems [1,2,3]. Owing to its high energy density and zero-carbon emissions, H is increasingly viewed as a promising alternative to conventional fossil fuels for large-scale energy storage and transportation [4,5]. Austenitic stainless steels (ASSs) are widely employed as structural materials in H infrastructures [6,7,8] due to their superior corrosion resistance, mechanical strength, and processability. However, under conditions of H exposure, cyclic loading, and elevated pressure, these alloys are prone to H ingress [9], which can significantly impair their mechanical integrity. Specifically, H uptake reduces ductility and fracture toughness, thereby enhancing susceptibility to hydrogen embrittlement (HE) [10,11,12].

Understanding H diffusion behaviour in ASSs is essential for evaluating their performance in H-containing environments. Owing to the FCC lattice structure of austenite, H transport in these steels exhibits high solubility but intrinsically low diffusivity [13,14,15]. Early studies have shown that, under isothermal conditions, H diffusivity is largely independent of concentration, suggesting that lattice properties exert a stronger influence on transport than concentration gradients [16]. Alloying elements can significantly alter H mobility by modifying the local atomic environment, introducing lattice distortions, and generating both reversible and irreversible trapping sites [17,18,19,20,21]. For instance, atomistic calculations by Bruzzoni et al. [22] have demonstrated that Cr addition in Fe–Cr alloys increases the solution energy and migration barrier of interstitial H, owing to the repulsive Cr–H interaction and the associated local lattice distortion, thereby reducing the intrinsic lattice diffusivity of H. In parallel, H permeation experiments by Peñalva et al. [23] revealed a systematic decrease in H permeability with increasing Cr content in Fe alloys, which was attributed to enhanced trapping induced by Cr-related elastic strain fields. Ni, although recognised for stabilising the austenitic phase, has a more contentious effect on H diffusivity. While some studies attribute a slight enhancement in diffusivity to reduced trapping site density and improved structural uniformity [17], others report that increasing Ni content either raises the diffusion energy barrier or has little effect [16,21]. Additionally, temperature variations can modulate the effectiveness of alloying-induced traps, influence the mobility of H within the lattice, and even trigger thermally activated processes that are negligible at lower temperatures [19,24]. These effects are not isolated but instead interact intricately. Unfortunately, the mechanisms governing H diffusion and mobility in ASSs, particularly under the combined influence of temperature and alloying elements, remain insufficiently understood.

The deformation behaviour of FeCrNi-based ASSs is governed by a balance between phase transformation and dislocation-mediated slip, both of which are highly sensitive to alloying composition [7,25], H concentration [26,27], and temperature [28]. For example, first-principles calculations by Li et al. [29] have shown that varying Cr/Ni ratios significantly alter the γ-surface and stacking-fault energy (SFE) across different compositional and thermal regimes. Alloys with lower Ni-equivalent content (SFE < 18 mJ/m^2^) tend to favour ε-martensitic transformation, while higher Ni content raises the SFE, promoting dislocation slip or deformation twinning instead. In situ and nanoindentation experiments on 21Cr–6Ni stainless steels reveal that H enhances slip planarity and lowers the stress required for dislocation nucleation, consistent with H-enhanced localised plasticity (HELP) theory [30]. Furthermore, temperature-dependent tensile tests on H-charged ASSs demonstrate that cryogenic conditions promote strain-induced α′-martensite formation, thereby exacerbating embrittlement [31,32,33], while elevated temperatures increase atomic mobility and suppress transformation, shifting the dominant deformation mechanism toward dislocation slip [34,35,36]. Collectively, these results highlight that the deformation behaviour of ASSs arises from the intricate interplay among composition, temperature, and H environment.

Despite considerable progress, existing atomistic studies on H-affected FeCrNi-based ASSs have generally examined H diffusion, SFE variation, phase transformation, or dislocation activity under separately varied alloying composition, temperature, or H concentration conditions [37,38]. However, their coupled influence on H transport and deformation-mode transition remains insufficiently quantified at the atomistic scale. In particular, it remains unclear how Cr and Ni contents regulate H migration barriers over a broad temperature range, how H concentration modifies SFE and defect nucleation, and how these factors jointly shift the dominant deformation mode between martensitic transformation and dislocation-mediated plasticity [39,40,41]. This knowledge gap can be quantitatively addressed through H diffusion coefficients, activation energies, SFE distributions, stress–strain responses, phase fractions, dislocation evolution, and vacancy formation under controlled combinations of Cr/Ni content, temperature, and H concentration. Experimental techniques face intrinsic limitations in capturing atomistic interactions and transient defect dynamics [42,43,44]. In this context, molecular dynamics (MD) simulations provide a powerful means to explore H–matrix interactions, defect evolution, and transformation mechanisms at the atomic scale [45,46], thereby delivering complementary insights that are challenging to obtain experimentally.

Accordingly, the novelty of the present study lies in the systematic integration of H diffusion analysis, statistical SFE evaluation, and tensile deformation simulations within a unified Fe–Cr–Ni–H interatomic-potential framework. Specifically, H diffusivity in FeCrNi-based ASSs was quantified by calculating diffusion coefficients and activation energies using mean square displacement (MSD) of H atoms and Arrhenius theory analysis across varying Cr and Ni contents and temperatures. Subsequently, tensile tests were performed on three representative alloying systems to evaluate the combined effects of alloying composition, H concentration, and temperature on the mechanical response and deformation mechanisms such as phase transformation and dislocation-mediated plasticity. Ultimately, H-enhanced dislocation plasticity assisted by temperature was linked with the formation of vacancies and H–vacancy complexes.

## 2. Results and Discussion

### 2.1. Effect of Alloying Elements and Temperature on H Diffusion Behaviour

**Figure 1:**
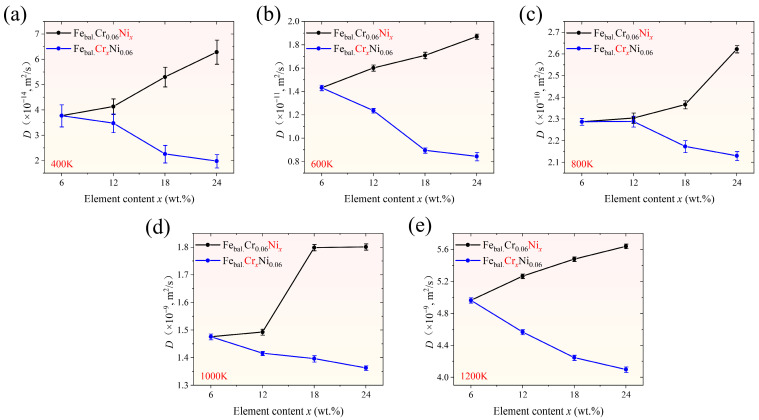
Diffusion coefficients of H in the monocrystalline models for varying Cr and Ni concentrations across all investigated temperatures: (**a**) 400 K, (**b**) 600 K, (**c**) 800 K, (**d**) 1000 K, and (**e**) 1200 K. The “Fe_bal._Cr_x_Ni_0.06_” denotes a model with a fixed Ni mass fraction of 0.06 and a variable Cr mass fraction, x = 0.06, 0.12, 0.18, or 0.24, with Fe making up the remainder.

In the simulations, the initial concentrations of the alloying elements Cr and Ni were both set at 6 wt.%. Each element was then varied individually from 6 wt.% to 24 wt.% in increments of 6 wt.% [47]. The H concentration of 3 at.% was adopted as a representative value, as it adequately captures the diffusion characteristics within the modelled conditions. The diffusion coefficients of H were calculated from the MSD of H atoms, and the corresponding results are shown in Figure 1, where the error bars represent the uncertainty associated with MSD slope fitting. Despite these fitting-related fluctuations, the H diffusion coefficient exhibits an overall increase with rising Ni concentration across all investigated temperatures, whereas increasing Cr concentration results in a reduction in the diffusion coefficient.

**Figure 2:**
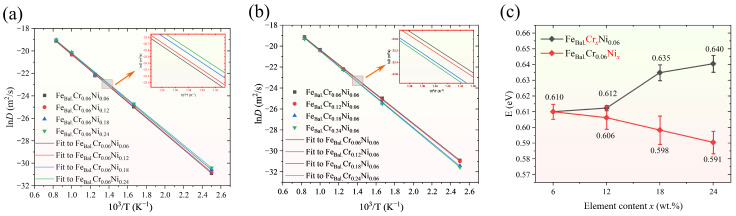
Diffusion coefficients of H in the (**a**) Fe_bal._Cr_0.06_Ni_x_ model and (**b**) Fe_bal._Cr_x_Ni_0.06_ model as a function of the reciprocal of temperature; (**c**) the activation energy E as a function of the alloying element content in all MD models.

Furthermore, the simulation results demonstrate that the H diffusion coefficient increases with increasing temperature. As shown in Figure 2a,b, there is a strong linear relationship between the diffusion coefficient and the reciprocal of temperature, in accordance with diffusion theory. Figure 2c presents the H diffusion activation energies (E), determined from the simulation results via the Arrhenius equation [13,48], as a function of alloying element content. It is evident that Cr increases the activation energy, whereas Ni decreases it in the Fe–Cr–Ni alloy system. For instance, relative to the Fe_bal._Cr_0.06_Ni_0.06_ model (=0.610 eV), the activation energy rises to 0.640 eV in the Fe_bal._Cr_0.24_Ni_0.06_ model and falls to 0.591 eV in the Fe_bal._Cr_0.06_Ni_0.24_ model, corresponding to an increase of 0.030 eV and a decrease of 0.019 eV, respectively. This suggests that Cr increases the energy barrier for H atom diffusion within the crystal lattice, thus hindering diffusion, whereas Ni lowers the barrier and facilitates diffusion. The activation energies obtained in this study (0.591–0.640 eV) are in close agreement with experimentally reported values for ASSs (0.529–0.611 eV) [16,19,49].

### 2.2. Effect of Alloying Elements on Tensile Deformation Mechanism of Fe–Cr–Ni Monocrystalline Models

**Figure 3:**
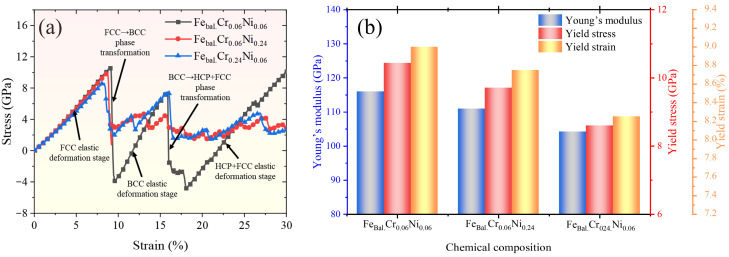
(**a**) Uniaxial tensile stress–strain curves of Fe_bal._Cr_0.06_Ni_0.06_, Fe_bal._Cr_0.06_Ni_0.24_, and Fe_bal._Cr_0.24_Ni_0.06_ models; (**b**) Young’s modulus and yield stress and strain as a function of chemical composition.

The stress–strain curves for the tensile deformation of the three monocrystalline models (Fe_bal._Cr_0.06_Ni_0.06_, Fe_bal._Cr_0.06_Ni_0.24_, and Fe_bal._Cr_0.24_Ni_0.06_) are presented in Figure 3a, with the corresponding mechanical property parameters summarised in Figure 3b. These compositions are selected as representative cases from the investigated composition space to illustrate the effect of Cr and Ni content on tensile deformation behaviour. At the initial stage of uniaxial tensile loading, all monocrystalline models exhibit typical elastic deformation behaviour. Notably, an increase in the Cr and Ni alloying contents leads to a reduction in both Young’s modulus and yield stress. In the present defect-free single crystal models, the yield point reflects a nucleation-controlled instability rather than conventional dislocation glide [50]. The reduction in the yield stress is therefore attributed to alloying-induced lattice distortion, which lowers the energetic barrier for phase transformation. Similarly, the decrease in Young’s modulus reflects the composition-dependent elastic response predicted by the present Fe–Cr–Ni–H potential for the selected crystal orientation and random solid-solution configurations. These values are thus used mainly to compare relative trends among models under identical simulation conditions, rather than to represent absolute mechanical properties of engineering ASSs.

Following the elastic deformation phase, monocrystalline models with various alloying element contents exhibit two distinct stress–strain behaviours. In terms of the model of Fe_bal._Cr_0.06_Ni_0.06_, the stress–strain curve shows a peak during initial FCC elastic deformation, followed by a drop into negative values. This is succeeded by a second elastic deformation stage, another transition into negative stress, and finally a third elastic stage. In contrast, the Fe_bal._Cr_0.06_Ni_0.24_ and Fe_bal._Cr_0.24_Ni_0.06_ models manifest a different response. After the initial elastic stage, plastic deformation becomes the dominant mode during the subsequent loading process.

**Figure 4:**
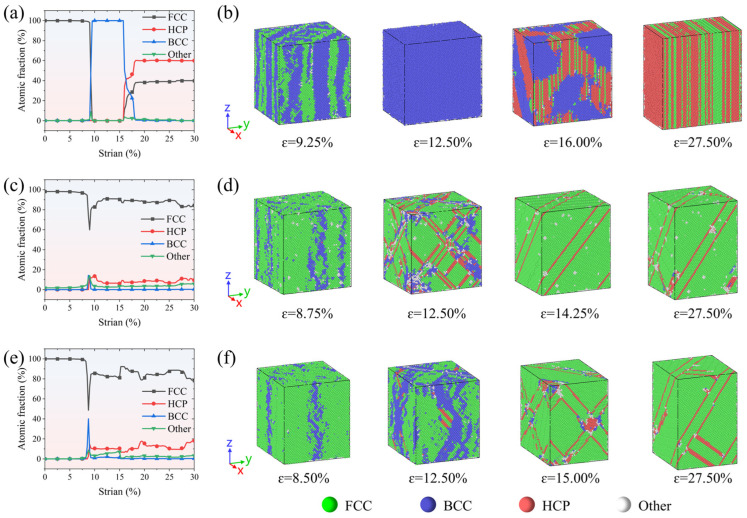
Atomic friction of various phases as a function of tensile strain and detailed MD snapshots of the plastic deformation mechanism for (**a**,**b**) Fe_bal._Cr_0.06_Ni_0.06_, (**c**,**d**) Fe_bal._Cr_0.06_Ni_0.24_, and (**e**,**f**) Fe_bal._Cr_0.24_Ni_0.06_ models. Red, green, blue, and white spheres represent HCP, FCC, BCC, and other crystal structures, as identified by common neighbour analysis (CNA).

The combined analysis of structural changes in FeCrNi-based ASSs during uniaxial tensile deformation, as shown in Figure 4, elucidates the origin of the observed negative stress phenomenon. During the initial elastic deformation stage, the increasing interatomic distance along the tensile direction leads to the deviation of atoms from equilibrium positions, enhancing interatomic attraction and thus increasing tensile stress. After the first stress peak, the resultant local stress concentration and lattice instability trigger a diffusion-less transformation from FCC to BCC. This transformation entails atomic rearrangement within the FCC lattice, characterised by compression along one crystallographic axis and elongation along another, ultimately resulting in a new equilibrium BCC phase, such as that observed at a strain of 12.50% in Figure 4b. The transition is accompanied by a weakening of atomic bonding and a sharp decrease in stress, occasionally even leading to negative stress values [51,52,53], which reflect rapid stress relaxation associated with abrupt lattice reconfiguration under defect-free and high-strain-rate conditions. Subsequently, the newly formed BCC martensite experiences elastic deformation up to a strain of 15.85%. Beyond this point, it begins to transform into the HCP phase, which is interpreted as a strain-induced martensitic transformation of the BCC phase rather than an equilibrium phase transition. Simultaneously, the accumulated strain drives some regions of the BCC phase to undergo an inverse transformation, reverting back to the FCC structure. As part of the deformation coordination mechanism, the newly formed HCP and reverted FCC phases align along the (010) planes, giving rise to an alternating lamellar structure. This characteristic organisation is exemplified at a strain of 27.50%, as shown in Figure 4b. This observation aligns with previously reported findings on phase transformations occurring during the uniaxial tension of FCC single crystal alloys [53].

**Figure 5:**
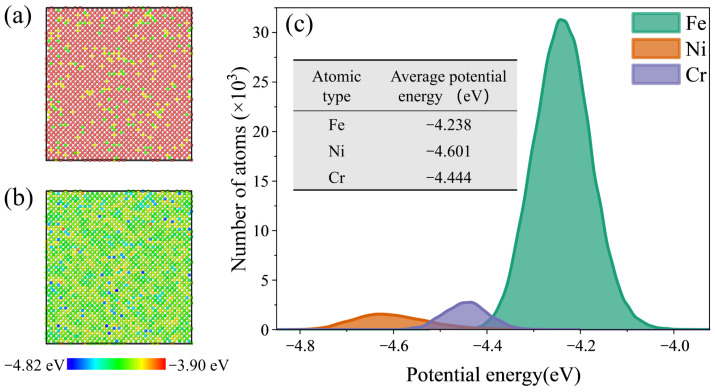
Cross-sectional view showing (**a**) the atomic distribution and (**b**) the per-atom potential energy in the Fe_bal._Cr_0.06_Ni_0.06_ model. (**c**) Normal distributions and average potential energies for Fe, Cr, and Ni.

For the Fe_bal._Cr_0.06_Ni_0.24_ and Fe_bal._Cr_0.24_Ni_0.06_ models, the characteristic FCC→BCC→HCP martensitic transformation disappears. Instead, dislocation slip becomes the dominant plastic deformation mechanism. This shift is likely attributable to the reduction in the system’s potential energy induced by the addition of Cr or Ni. As illustrated in Figure 5b, the potential energies of Cr and Ni atoms are markedly lower than those of the Fe matrix. To enable a more detailed quantitative analysis, the normal distributions and average per-atom potential energies for Fe, Cr, and Ni are statistically summarised in Figure 5c. Clearly, Cr and Ni atoms exhibit lower average per-atom potential energy (−4.444 eV and −4.601 eV) compared to Fe atoms (−4.238 eV), thereby lowering the overall potential energy of the FeCrNi-based ASSs. This reduction in system energy enhances the thermodynamic stability of the austenitic phase, thereby effectively suppressing its transformation to BCC and HCP martensite during tensile deformation. As a consequence, the dominant deformation mode in the Fe_bal._Cr_0.06_Ni_0.24_ and Fe_bal._Cr_0.24_Ni_0.06_ models shift from transformation-mediated deformation to dislocation-mediated plasticity, which is mainly accommodated by the nucleation and glide of Shockley partial dislocations on close-packed planes within the FCC matrix.

**Figure 6:**
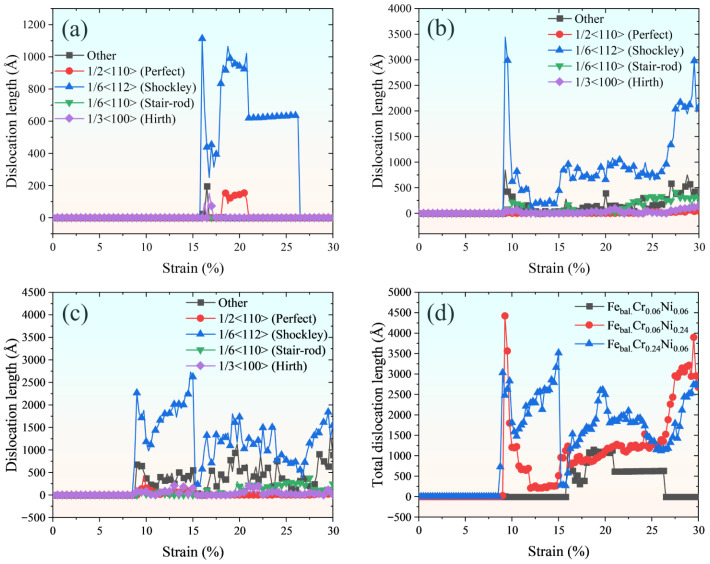
(**a**–**c**) Evolution of various types of dislocations as a function of tensile strain during uniaxial loading for Fe_bal._Cr_0.06_Ni_0.06_, Fe_bal._Cr_0.06_Ni_0.24_, and Fe_bal._Cr_0.24_Ni_0.06_ models, respectively. (**d**) Evolution of the total dislocation length as a function of tensile strain during uniaxial loading for the three models. Perfect, Shockley, stair-rod, Hirth, and other types of dislocations are identified using the dislocation extraction algorithm (DXA) and are included in the statistical analysis of total dislocation length.

**Figure 7:**
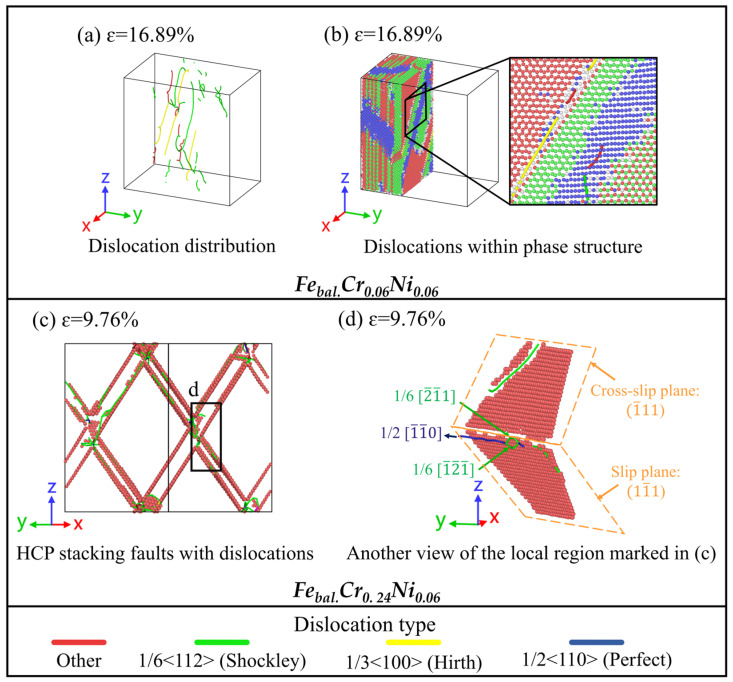
Atomic configurations of the phase transition and dislocation activity in the (**a**,**b**) Fe_bal._Cr_0.06_Ni_0.06_ and (**c**,**d**) Fe_bal._Cr_0.24_Ni_0.06_ monocrystalline models at different tensile strains. Matrix atoms are partially hidden or sectioned in selected panels for clarity. Red, green, blue, and white spheres represent HCP, FCC, BCC, and other crystal structures, respectively, as identified by CNA. Green, yellow, blue, and red lines represent Shockley, Hirth, Perfect, and other types of dislocations.

Figure 6 presents a statistical analysis of the evolution of dislocation length during uniaxial tensile loading for the investigated models. As shown in Figure 6a–c, Shockley partial dislocations are the predominant type across all three models. This is consistent with the FCC deformation mode, in which Shockley partials can nucleate on close-packed slip planes and leave stacking faults (SFs) during glide. As illustrated in Figure 6d, compared to the Fe_bal._Cr_0.06_Ni_0.06_, increasing the Cr or Ni alloying contents leads to a pronounced increase in dislocation length, indicating more active dislocation nucleation and propagation during tensile deformation. This is in concurrence with the aforementioned reduction in yield stress caused by Cr or Ni additions, as shown in Figure 3. Figure 7b further illuminates the dislocation activity of the Fe_bal._Cr_0.24_Ni_0.06_ model. At the early stages of plastic deformation, numerous SFs form on close-packed planes, originating from the nucleation and glide of Shockley partial dislocations, which serve as the primary carriers of localised plasticity. Figure 7c provides an enlarged view of a selected region from Figure 7b, revealing atomic-scale details of dislocation interactions. Two Shockley partial dislocations with Burger’s vector $1/6[\bar{2}\bar{1}1]$ and $1/6[\bar{1}\bar{2}\bar{1]}$ initially glide on the ($1\bar{1}1$) plane. As deformation proceeds, these partial dislocations undergo cross-slip onto the intersecting ($1\bar{1}1$) plane. To facilitate this process, two partials are first encountered on the intersecting slip planes and subsequently merge into a screw dislocation. Due to the high crystallographic symmetry of FCC structures, this screw dislocation can glide across multiple planes. The specific reaction is as follows:

(1) $$1/6[\bar{2}\bar{1}1]+1/6[\bar{1}\bar{2}\bar{1]}\to 1/2[\bar{1}\bar{1}0]$$

This dislocation reaction generates a perfect screw dislocation, illustrating a characteristic transformation pathway as predicted by the Thompson tetrahedron model. It exemplifies the cross-slip mechanism, in which partial dislocations sequentially switch slip planes and interact with one another. This process facilitates dislocation multiplication and plays a key role in sustaining plastic deformation.

### 2.3. Effect of H Concentration on Tensile Deformation Mechanism of Fe–Cr–Ni Monocrystalline Models

**Figure 8:**
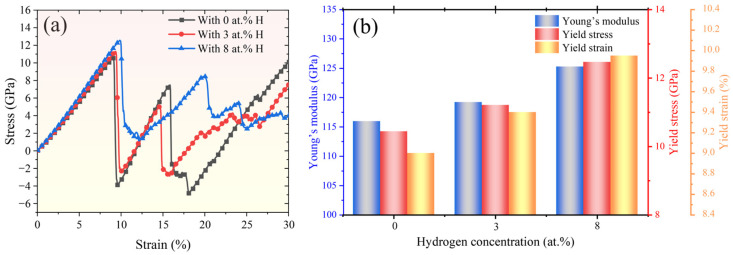
(**a**) Uniaxial tensile stress–strain curves of the Fe_bal._Cr_0.06_Ni_0.06_ model with 0 at.%, 3 at.%, and 8 at.% H; (**b**) Young’s modulus and yield stress and strain as a function of H concentration.

**Figure 9:**
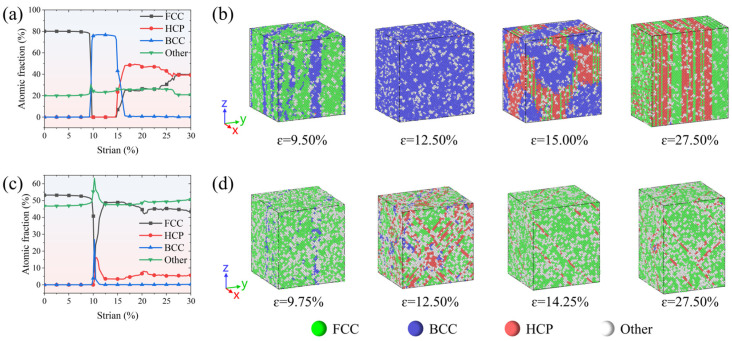
Atomic friction of various phases as a function of tensile strain and detailed MD snapshots of the plastic deformation mechanism for the Fe_bal._Cr_0.06_Ni_0.06_ model with (**a**,**b**) 3 at.% H and (**c**,**d**) 8 at.% H. Red, green, blue, and white spheres represent HCP, FCC, BCC, and other crystal structures, as identified by CNA.

A series of simulations were carried out to investigate the effect of H concentration on the deformation mechanisms of various FeCrNi-based ASSs. In this part of the study, the Fe_bal._Cr_0.06_Ni_0.06_ monocrystalline model was selected as a representative system to investigate how H concentrations of 0 at.%, 3 at.%, and 8 at.% affect its tensile deformation behaviour, as this composition provides a compositional baseline within the investigated alloy space and avoids additional complexity arising from variations in Cr and Ni contents. As shown in Figure 8a, the stress–strain curves of both the H-free system and the system with a low H concentration of 3 at.% exhibit similar mechanical responses, indicating an FCC→BCC→HCP martensitic transformation, as shown in Figure 9. In contrast, the tensile behaviour of the model with a higher H concentration of 8 at.% reveals a suppressed phase transformation and a pronounced enhancement in dislocation slip activity. This transition indicates that high H concentration changes the dominant deformation carrier from transformation-mediated deformation to dislocation-mediated plasticity. Further quantitative analysis presented in Figure 8b indicates that increasing H concentration significantly enhances the mechanical properties of the FCC austenite matrix. Specifically, raising the H concentration from 0 to 8 at.% leads to an approximate 7.45% increase in yield stress, accompanied by a rise in yield strain from 9.00% to 9.50%. The observed increase in yield stress can be attributed to the H-induced solid solution, as reported by Ogawa et al. [8], who experimentally investigated HE behaviour in ASSs.

**Figure 10:**
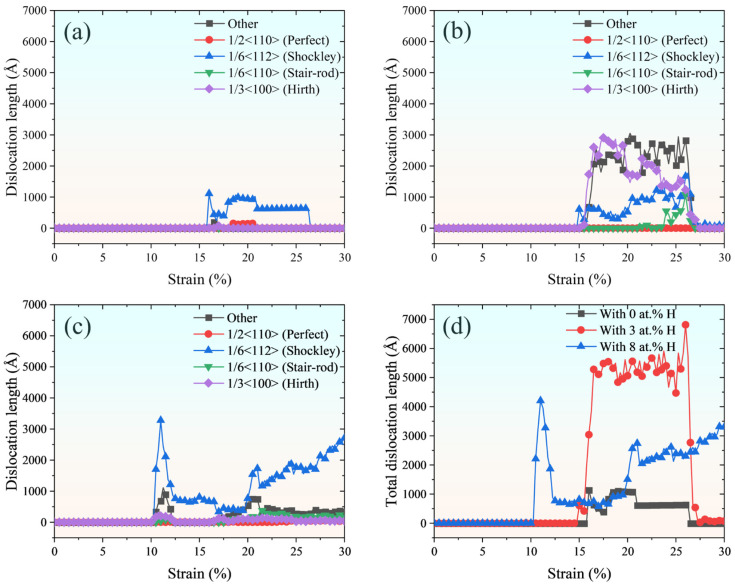
(**a**–**c**) Evolution of various types of dislocations as a function of tensile strain during uniaxial loading for the Fe_bal._Cr_0.06_Ni_0.06_ model with H concentrations of 0 at.%, 3 at.%, and 8 at.%, respectively. (**d**) Evolution of the total dislocation length as a function of tensile strain during uniaxial loading for the three models. Perfect, Shockley, stair-rod, Hirth, and other types of dislocations are identified using the DXA and included in the statistical analysis of total dislocation length.

**Figure 11:**
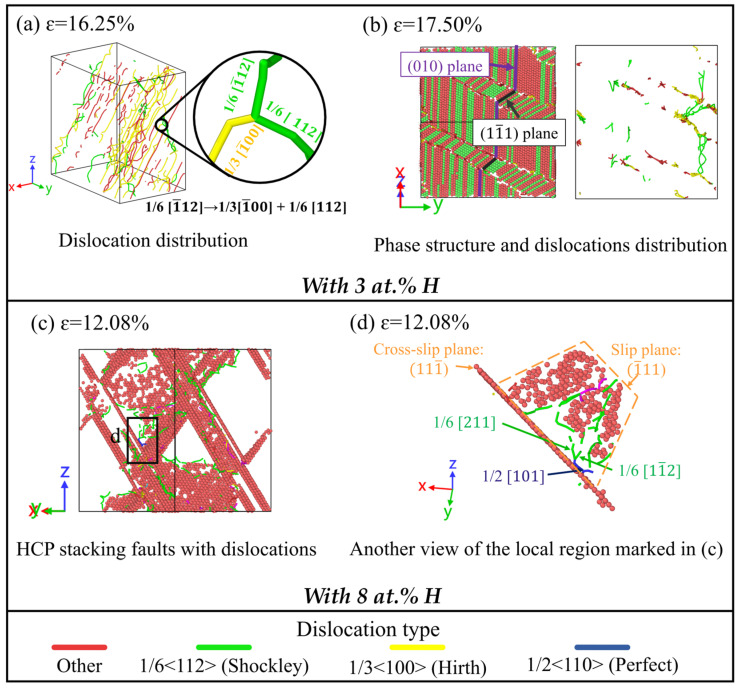
Atomic configurations of the phase transition and dislocation activity in the Fe_bal._Cr_0.06_Ni_0.06_ model with (**a**,**b**) 3 at.% H and (**c**,**d**) 8 at.% H. Matrix atoms are partially hidden or sectioned in selected panels for clarity. Red, green, blue, and white spheres denote HCP, FCC, BCC, and other crystal structures, respectively, as identified by CNA. Green, yellow, blue, and red lines represent Shockley, Hirth, Perfect, and other types of dislocations.

Based on the DXA analysis, Figure 10 shows that the alloying system with 3 at.% H exhibits a higher dislocation length compared to the H-free counterpart. Furthermore, the atomic-scale analysis in Figure 11a reveals that the model containing 3 at.% H displays a broader range of dislocation types, including 1/3<100> Hirth, 1/6<110> stair-rod, and other dislocation configurations formed at SF intersections. This increased dislocation diversity is attributed to H-induced lattice distortions, which promote dislocation multiplication and enhance interactions among dislocations. For instance, the Hirth dislocations can result from reactions of Shockley dislocations as follows:

(2) $$1/6\left[\bar{1}12\right]\to 1/3\left[\bar{1}00\right]+1/6\left[112\right]$$

It is also noteworthy that while an FCC→BCC→HCP martensitic transformation occurs at a H concentration of 3 at.%, similar to the H-free case, the phase transition pathways differ. In the absence of H, the BCC-to-HCP phase transition proceeds exclusively along the (010) crystallographic plane. However, with the introduction of 3 at.% H atoms, additional phase transition pathways are activated along both the (010) and ($1\bar{1}1$) crystallographic planes, as shown in Figure 11b. The involvement of the ($1\bar{1}1$) plane introduces a complementary shear mechanism, creating intersections with the (010) plane that facilitate the nucleation of Hirth and other dislocations, thereby enhancing overall dislocation activity. At a H concentration of 8 at.%, the FCC→BCC→HCP phase transition is not observed. Instead, pronounced localised dislocation plasticity is directly reflected by the DXA-identified dense and short Shockley partial dislocations, as shown in Figure 10c and Figure 11c.

**Figure 12:**
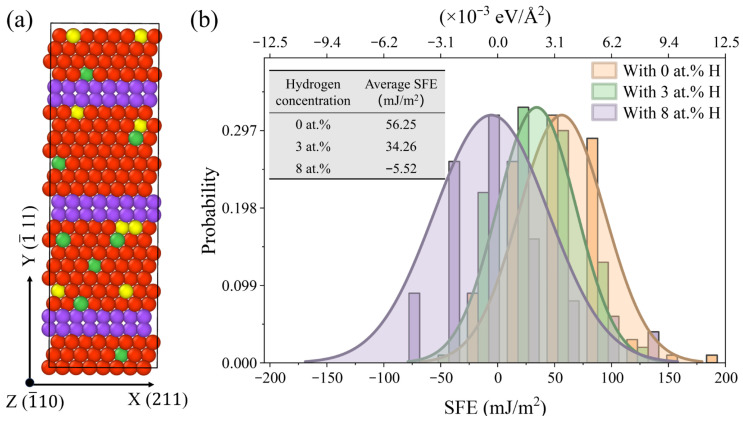
(**a**) Illustration of the computational cell including three SF planes, as indicated by purple atoms. (**b**) The SFE statistics for the Fe_bal._Cr_0.06_Ni_0.06_ model with varying H concentrations. Red, green, and yellow atoms represent Fe, Ni, and Cr, respectively.

As discussed above, the addition of H can suppress the phase transition and encourage dislocation-mediated plasticity. Consistent experimental evidence on H–dislocation interactions in FCC alloys has shown that H markedly enhances dislocation activity and promotes planar slip behaviour [54]. It is well known that dislocation nucleation and propagation are closely related to the SFE [55], and the SFE is H concentration-dependent. As such, additional atomistic simulations were performed to illuminate the evolution of SFE as a function of H concentration. A representative slip system was selected, and three SF planes were introduced by displacing the crystal along the Burgers vector at three equally spaced positions along the Y-axis, as demonstrated in Figure 12a. The SFE was calculated as the energy difference between the faulted and unfaulted systems at various H concentrations, normalised by the total fault area. To statistically capture the effects of local alloying composition and H interstitial locations, 1800 random seeds were used to generate 1800 individual SFE values. The reported SFE value therefore represents a statistical average over the sampled local configurations, rather than a single local SFE value. The resulting SFE distributions and running averages were then computed and are presented in Figure 12b.

As shown, H atoms clearly shift the SFE distribution to lower values, indicating a reduction in the average SFE with increased H concentration. At a low H concentration (3 at.%), the SFE decreases moderately. As the SFE remains relatively high, the FCC→BCC →HCP martensitic transformation still takes place. However, the addition of H induces an additional phase transition pathway and promotes the nucleation of Hirth and other types of dislocations. At a H concentration of 8 at.%, the SFE is markedly reduced to −5.52 mJ/m^2^, as determined from the overall SFE distribution. At this concentration, the SFE exhibits pronounced spatial heterogeneity, with both positive and negative values resulting from locally varying H environments. The negative average SFE indicates that SF formation becomes energetically favourable, reflecting strong local instability of the FCC stacking sequence. As a result, SFs can nucleate and propagate within H-enriched regions. This pronounced reduction in SFE strongly favours dislocation-mediated plasticity, while phase transformations become increasingly unfavourable or are even completely suppressed. Because SFs in FCC crystals are generated by the glide of Shockley partial dislocations, the reduced SFE facilitates frequent partial-dislocation nucleation in H-enriched regions and promotes localised SF formation. The enhanced tendency for SF formation is consistent with the dense and short Shockley partial dislocations observed in Figure 10c and Figure 11c, which reflect localised dislocation-mediated plasticity rather than long-range martensitic transformation. The enhanced dislocation activity, stacking-fault formation, and localised slip induced by H are therefore interpreted primarily within the framework of the HELP mechanism. In contrast, the present simulations do not explicitly model crack-tip decohesion or interatomic bond separation associated with hydrogen-enhanced decohesion (HEDE). Since the current monocrystalline models mainly focus on H-assisted dislocation activity, phase transformation, and vacancy evolution during tensile deformation, the observed H effects are more directly related to HELP-assisted plasticity and vacancy-mediated damage evolution rather than pure decohesion-controlled fracture.

### 2.4. H-Induced Dislocation Plasticity Assisted by Temperature

**Figure 13:**
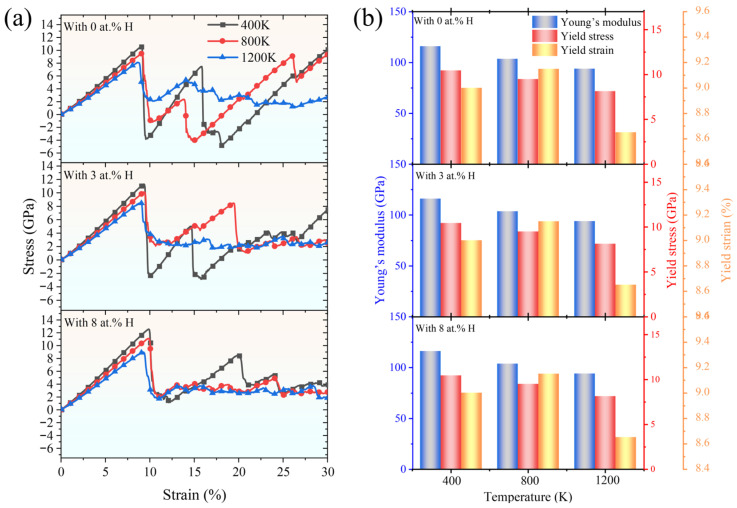
(**a**) Uniaxial tensile stress–strain curves of the Fe_bal._Cr_0.06_Ni_0.06_ model with 0 at.% H, 3 at.% H, and 8 at.% H at 400 K, 800 K, and 1200 K; (**b**) Young’s modulus and yield stress and strain as a function of temperature.

To demystify the thermal dependence of H-induced dislocation plasticity, the tensile stress–strain curves of FeCrNi-based ASSs charged with varying H concentrations of 0 at.%, 3 at.%, and 8 at.% at temperatures of 400 K, 800 K, and 1200 K are included in Figure 13. As the temperature rises, the yield stress exhibits a decrease, primarily attributed to the thermal weakening of atomic bonding forces [56,57], which diminishes the material’s resistance to plastic deformation. Notably, the coupled effects of temperature and H concentration exert a pronounced influence on both the phase transformation behaviour and the predominant plastic deformation mechanism. As shown in Figure 13a, in the absence of H, elevated temperatures alone can suppress the FCC→BCC→HCP martensitic transformation. A pronounced stress drop, characteristic of this transformation, is evident at 400 K and 800 K but vanishes at 1200 K. With 3 at.% H, the transformation is already absent at 800 K, and with 8 at.% H, it is suppressed at all examined temperatures. These results indicate that increasing temperature and H concentration jointly favour dislocation-mediated plasticity and inhibit martensitic transformation, which is consistent with the enhanced dislocation activity observed at elevated temperatures and higher H contents.

**Figure 14:**
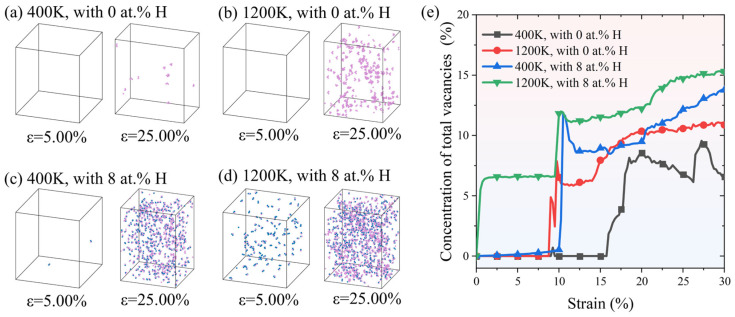
The formation of vacancies at tensile strains of ε = 5.00% and ε = 25.00% for the model with various temperatures and H concentrations: (**a**) 400 K with 0 at.% H, (**b**) 1200 K with 0 at.% H, (**c**) 400 K with 8 at.% H, and (**d**) 1200 K with 8 at.% H. (**e**) Corresponding evolution of the concentration of total vacancies as a function of tensile strain, quantified using WS analysis. Pink spheres denote vacancies, and blue spheres indicate H atoms.

It has been revealed by experiments and simulations that dislocation plasticity generates vacancies [58,59], and those vacancies preserved by H have implications on H-induced failure [60,61]. Inspired by this, we calculated the evolution of vacancies during the tensile deformation process by using Wigner–Seitz (WS) defect analysis. As shown in Figure 14a, the Fe_bal._Cr_0.06_Ni_0.06_ model devoid of H at 400 K exhibits few vacancies, even at a high strain of 25%. Increasing temperature enhances dislocation-mediated plasticity and thus generates more vacancies, for example, at 1200 K, as shown in Figure 14b. Although the high strain rate employed in the simulations may influence vacancy formation, a pronounced dependence on H concentration is still clearly observed. In contrast, the introduction of 8 at.% H, even at a relatively low temperature of 400 K, markedly increases the number of vacancies. On the one hand, H facilitates the cross-slip of dislocations, as shown in Figure 11d. This process contributes to the spatial distribution of vacancies, consistent with previous reports that vacancies mainly originate from four dislocation processes: the growth of prismatic loops, non-conservative jog dragging, cross-slip of dislocations, and the breaking of dislocation junctions [62]. On the other hand, density functional theory (DFT) calculations show that H can lower both the formation energy of vacancies and bind strongly with vacancies to form the H–vacancy complexes [63,64]. Therefore, vacancy-type defects can act as favourable sites for H stabilisation, and the formation of H–vacancy complexes provides a defect-level mechanism for vacancy retention during deformation. Vacancy complexes are stabilised, and Figure 14e indicates that H promotes their growth through dislocation interactions, leading to a pronounced increase in vacancy concentration at higher tensile strain. As such, the concentration of total vacancies can be further amplified by high temperature and H concentration, as shown in Figure 14d.

The increase in vacancy concentration plays a critical role in the mechanical behaviour of ASSs. From the perspective of vacancy-mediated H damage, H–vacancy complexes provide a direct link between H trapping at vacancy-type defects and subsequent defect accumulation. In H-enriched regions, H–vacancy complexes facilitate vacancy clustering, which, under sustained plastic deformation, can evolve into proto-nanovoids [65,66]. These nanoscale voids act as stress concentrators and disrupt cohesive bonding along slip bands and grain boundaries, and further lead to macroscopic failure by void growth and coalescence processes, in concordance with experimental evidence for nanovoiding in H-induced embrittled ASSs along quasi-brittle fracture surfaces [67,68,69]. Therefore, the present vacancy evolution results support a vacancy-mediated damage pathway, in which H–vacancy interactions stabilise vacancy-type defects, promote vacancy clustering, and facilitate proto-nanovoid formation during continued deformation. In H-containing environments, thermal fluctuations and stress concentrations at interfaces can further promote vacancy clustering and void formation [65,70,71]. The combined influence of elevated temperatures and local H enrichment accelerates vacancy clustering and void growth, ultimately reducing the fatigue resistance and fracture life of ASS components in service.

## 3. Computational Methodology

All MD simulations were performed using the Large-Scale Atomic/Molecular Massively Parallel Simulator (LAMMPS) code [72]. LAMMPS is a classical MD simulation package designed for large-scale particle-based modelling and is particularly suitable for parallel simulations involving short-range interatomic interactions. To investigate the effect of alloying elements Ni and Cr on H diffusion and mechanical response of ASSs, the recently improved embedded-atom method (EAM) potential for the Fe–Cr–Ni–H system by Zhou et al. [73] was employed. The EAM is a semi-empirical potential formalism commonly used for metallic systems, in which the total energy of the system is determined by both pairwise atomic interactions and the embedding energy associated with the local electron density contributed by neighbouring atoms. This framework is suitable for describing metallic bonding, defect structures, and mechanical responses in metal and alloy systems [74]. The Fe–Cr–Ni–H EAM potential developed by Zhou et al. is capable of capturing the correct H interstitial site, swelling volume, diffusion energy barrier, relative energies between H and various metal elements, and interaction energies between H and point defects, and is thus considered acceptable for our purposes.

**Figure 15:**
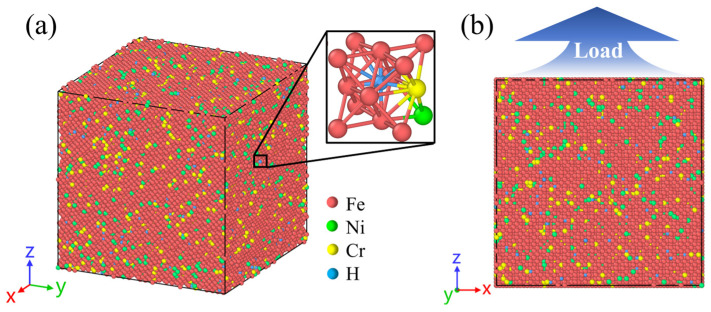
(**a**) Initial configuration of Fe–Cr–Ni–H single crystal, with a localised enlargement showing the octahedral sites occupied by H atoms within the FCC matrix, and (**b**) schematic of the uniaxial tensile loading. Red, green, yellow, and blue spheres represent Fe, Ni, Cr, and H atoms, respectively.

A single crystal Fe–Cr–Ni model was adopted to enable a clear interpretation of composition- and H-dependent behaviour, providing a foundation for future extensions to polycrystalline systems. The present monocrystalline FCC models do not explicitly include grain boundaries, secondary phases, or microstructural heterogeneity. Therefore, grain-boundary-mediated H trapping, intergranular diffusion, deformation incompatibility, and grain-boundary-assisted cracking mechanisms are beyond the scope of the current study. The purpose of the simplified monophasic model is to isolate the intrinsic atomistic effects of alloying composition, temperature, and H concentration on H diffusion, stacking-fault stability, phase transformation, and dislocation activity. The FCC Fe single crystal has dimensions of 109.3 Å × 109.3 Å × 109.3 Å, and orientations such that the X-, Y-, and Z-axes correspond to the [100], [010], and [001] crystallographic directions, respectively. Periodic boundary conditions were applied in all three directions. To mimic FeCrNi-based ASSs, Fe atoms in the supercell were randomly substituted for Cr and Ni atoms based on the target atomic fractions (hereafter referred to as at.%), which were calculated from corresponding mass fractions (hereafter referred to as wt.%). It should be noted that possible short-range order was not explicitly introduced in the present random solid-solution model. Since short-range order may modify the local chemical environment around interstitial H atoms and thus influence H mobility, its effect will be further examined in future work. Numerous studies have examined the microstructural evolution of ASSs over a wide range of Cr and Ni mass fractions, typically from 2 wt.% to 25 wt.% [27,47,75,76,77]. Accordingly, the present study conducted a detailed analysis of Cr and Ni mass fractions of 6 wt.%, 12 wt.%, 18 wt.%, and 24 wt.%. For the H diffusion simulations, Fe_bal._Cr_x_Ni_0.06_ and Fe_bal._Cr_0.06_Ni_x_ denote two model series in which one alloying element was fixed at a mass fraction of 0.06 while the other varied as x = 0.06, 0.12, 0.18, or 0.24, with Fe making up the balance. For the tensile simulations, Fe_bal._Cr_0.06_Ni_0.06_, Fe_bal._Cr_0.06_Ni_0.24_, and Fe_bal._Cr_0.24_Ni_0.06_ were selected to compare the effects of increased Ni or Cr content. H atoms were created at concentrations of 0 at.%, 3 at.%, and 8 at.% [51,77], randomly occupying octahedral interstitial sites within the model. The Fe–Cr–Ni–H structures were then equilibrated under the isothermal–isobaric (NPT) ensemble by heating to 1200 K for 1 ns with a timestep of 0.001 ps, followed by cooling to 400 K over 0.5 ns, to promote H redistribution, minimise the system energy, and ensure atomic stability. The resulting relaxed Fe–Cr–Ni–H single crystal model is illustrated in Figure 15. To investigate the influence of temperature on H diffusion and HE, simulations were performed at 400 K, 600 K, 800 K, 1000 K, and 1200 K [73,78,79].

The H diffusion coefficient can be determined by analysing the typical MSD, which is defined as follows [80]:

(3) $$D=\underset{t\to \infty }{\lim}\frac{1}{6N}\sum_{i=1}^{N}{\left[\vec{r_{i}}\left(t+t_{0}\right)-\vec{r_{i}}\left(t_{0}\right)\right]}^{2}$$

where $\frac{1}{N}\sum_{i=1}^{N}{\left[\vec{r_{i}}\left(t+t_{0}\right)-\vec{r_{i}}\left(t_{0}\right)\right]}^{2}$ is the MSD of H atoms, *N* is the total number of H atoms, $\vec{r_{i}}\left(t_{0}\right)$ is the initial position of atom i, and $\vec{r_{i}}\left(t+t_{0}\right)$ is the position of atom i at time *t*.

In the mechanical property simulations, the relaxed model was subjected to uniaxial tensile loading along the *Z*-axis at a constant strain rate of 5 × 10^9^ s^−1^ [46,81], with the maximum strain reaching 30.00%. Due to the intrinsic time-scale limitation of classical MD simulations, this relatively high strain rate was applied consistently to all models, allowing the relative mechanical responses among different alloy compositions and H concentrations to be compared under the same simulation framework. Under this condition, long-range H diffusion may be kinetically limited, while short-range H–lattice and H–defect interactions remain active and allow the atomistic features of H-related deformation to be analysed. The simulation results were post-processed using OVITO [82], an open visualisation and analysis tool for atomistic simulation data. Specifically, the dislocation extraction algorithm (DXA) implemented in OVITO was used to identify dislocation structures and classify them according to their Burgers vectors, including Perfect, Shockley partial, stair-rod, Hirth, and other dislocations. The evolution of the total and type-specific dislocation lengths was then used to quantify dislocation activity during tensile deformation. WS defect analysis was used to characterise vacancy evolution. In addition, CNA was employed to monitor changes in the crystal structure and to identify phase transitions within the model.

## 4. Conclusions

In this study, atomistic simulations are employed to systematically investigate the effects of alloying element content and temperature on H diffusion in FeCrNi-based ASSs. Further, the influence of alloying composition, temperature, and H concentration on the evolution of mechanical properties and plastic deformation mechanisms is demystified. The key findings are summarised as follows:

1. Cr and Ni exert opposite effects on H transport by altering the migration energy landscape in the FCC matrix. Cr increases the activation energy for H migration and suppresses H mobility, whereas Ni lowers the activation barrier and facilitates H transport. Meanwhile, H diffusivity follows Arrhenius-type temperature dependence, indicating that temperature remains the dominant factor accelerating H migration in FeCrNi-based ASSs.

2. Alloying composition controls the intrinsic deformation mode by changing the stability of the FCC lattice. At low Cr and Ni contents of 6 wt.%, tensile loading induces sequential FCC→BCC→HCP martensitic transformations with characteristic stress drops. Increasing either Cr or Ni content to 24 wt.% stabilises the FCC phase, suppresses transformation, and shifts plasticity toward cross-slip-dominated dislocation activity.

3. H concentration modifies deformation behaviour primarily by reducing SF stability. At a low H content of 3 at.%, martensitic transformation is retained but proceeds through additional transformation pathways with enhanced dislocation diversity. At a high H concentration of 8 at.%, the SFE decreases significantly, martensitic transformation is suppressed, and plasticity is dominated by slip-mediated, HELP-like mechanisms characterised by dense Shockley partial dislocations.

4. Temperature and H concentration jointly promote vacancy-assisted damage by enhancing dislocation-mediated plasticity. Elevated temperature and high H content suppress martensitic transformation, intensify dislocation activity, and accelerate vacancy formation. H further stabilises vacancies through H–vacancy complexes, facilitating vacancy clustering and nanoscale void formation, which may contribute to H-assisted failure in FeCrNi-based ASSs.

## Data Availability

The original contributions presented in this study are included in the article. Further inquiries can be directed to the corresponding author(s).

## Abbreviations

The following abbreviations are used in this manuscript:

Austenitic stainless steels	ASSs
Hydrogen embrittlement	HE
Hydrogen	H
Stacking-fault energy	SFE
Hydrogen-enhanced localised plasticity	HELP
Hydrogen-enhanced decohesion	HEDE
Molecular dynamics	MD
Mean square displacement	MSD
Common neighbour analysis	CNA
Dislocation extraction algorithm	DXA
Stacking faults	SFs
Wigner–Seitz	WS
Density functional theory	DFT
Large-Scale Atomic/Molecular Massively Parallel Simulator	LAMMPS
Embedded-atom method	EAM
Open visualisation tool	OVITO
